# The Efficient Mobile Management Based on Metaheuristic Algorithm for Internet of Vehicle

**DOI:** 10.3390/s22031140

**Published:** 2022-02-02

**Authors:** Shih-Yun Huang, Shih-Syun Chen, Min-Xiou Chen, Yao-Chung Chang, Han-Chieh Chao

**Affiliations:** 1 Department of Electrical Engineering, National Dong Hwa University, Hualien 97401, Taiwan; 810523003@gms.ndhu.edu.tw; 2 Department of Computer Science and Information Engineering, National Dong Hwa University, Hualien 97401, Taiwan; 610621215@gms.ndhu.edu.tw (S.-S.C.); mxchen@gms.ndhu.edu.tw (M.-X.C.); 3 Department of Computer Science and Information Engineering, National Taitung University, Taitung 95092, Taiwan; ycc.nttu@gmail.com

**Keywords:** IoV, mobile management, loading balance, heuristic algorithm

## Abstract

With the low latency, high transmission rate, and high reliability provided by the fifth-generation mobile communication network (5G), many applications requiring ultra-low latency and high reliability (uRLLC) have become a hot research topic. Among these issues, the most important is the Internet of Vehicles (IoV). To maintain the safety of vehicle drivers and road conditions, the IoV can transmit through sensors or infrastructure to maintain communication quality and transmission. However, because 5G uses millimeter waves for transmission, a large number of base stations (BS) or lightweight infrastructure will be built in 5G, which will make the overall environment more complex than 4G. The lightweight infrastructure also has to be considered together. For these reasons, in 5G, there are two mechanisms for handover, horizontal, and vertical handover; hence, it must be discussed how to handle handover to obtain the best performance for the whole network. In this paper, to address handover selection, we consider delay time, energy efficiency, load balancing, and energy consumption and formulate it as a multi-objective optimization (MOO) problem. At the same time, we propose the handover of the mobile management mechanism based on location prediction combined with heuristic algorithms. The results show that our proposed mechanism is better than the distance-based one for energy efficiency, load, and latency. It optimizes by more than about 20% at most.

## 1. Introduction

In recent years, due to the development of smart devices and applications, people’s lives have become more convenient, and economic growth has also been promoted. There are many applications with a smart device such as an autopilot system, virtual reality (VR), and NB-IoT. Although these applications have become the focus of future technology development, they rely more on data transmission and real-time requests. To face these problems, 5G is an important technique. For the performance of 5G, the 3rd Generation Partnership Project (3GPP) has defined the many standards to achieve the goal [1,2,3]. Obtaining more bandwidth through higher frequency bands is one way to achieve the high capacity, low latency, and MIMO needs for 5G requirements. Since the coverage for the base station (BS) becomes smaller, most researchers think one of the promising solutions is to deploy lightweight infrastructures to support the transmission. According to the architecture of 5G, the network environment can include many different types of networks, such as ultra-dense network (UDN) [4], machine to machine communication (M2M), and Internet of Things (IoT). Hence, the 5G will be a heterogeneous network (HetNet) architecture that we can know, and it will complex than the traditional cellular network.

The IoV is a critical issue in 5G. Based on 5G and Internet of Things (IoT) technologies, the traditional vehicle ad hoc network (VANET) and vehicle-to-everything (V2X) will be integrated into the Internet of Vehicles (IoV) [5,6]. There are some types of communication in the V2X. They are vehicle to vehicle (V2V), vehicle to infrastructure (V2I), and vehicle to pedestrian (V2P), respectively [7]. Following the 3GPP standard, the V2X should fit the requirement with low latency and high reliability. According to [8], we know that IoV communication can be assisted by BSs/lightweight infrastructure and that the overall transmission can be faster and more reliable, considering that the 5G has the disadvantage for transmission range. At the same time, the vehicle user equipment (VUE) may access the BSs/lightweight infrastructures frequency which means the loading for each infrastructure will not balance. Considering the deployment of lightweight infrastructure is wide-range in 5G, handover of the mobile management strategy is different from the traditional cellular network. In traditional cellular wireless networks, handover only occurs between the two nearest BSs; however, in 5G, since it uses millimeter waves for transmission, it will result in rapid signal degradation when encountering buildings or obstacles. Therefore, handover between BSs alone is not feasible. According to [9], we can know that there will be two handover modes in 5G, horizontal and vertical handover. If the level of the next infrastructure is the same, it is called a horizontal handover; otherwise, when the target infrastructure is of a different level, a vertical handover occurs. Among them, vertical handover will be the biggest difference from 4G. When the handover is triggered, the BSs/lightweight infrastructures will send the information to the next infrastructure through signaling [10,11]. There are three phases for the process of handover. They are handover preparation, handover execution, and handover completion, respectively. First, the mobility management mechanism will measure the signal power of other accessible BSs/lightweight infrastructure and decide the handover strategy. Then, the original BS/light infrastructure will send the handover request to the target base station and process the registration when the handover strategy has been decided. At the same time, it also sends the VUE information to the buffer of the target BS. When the target BS receives the information from VUE, the radio resource control will build, and the BS starts the service for VUE. It also responds to the handover request that connects with the original BS will break if needed.

There is much literature on the handover strategy for mobile management. Recently, almost all handover trigger mechanisms follow the reference symbol received power (*RSRP*) [12,13] or received signal strength (*RSS*) [14]. These works also defined the threshold as $RSS_{th}$ and $RSRP_{th}$. By defining the threshold, it can avoid the ping-pong handover happening [15], and it can also solve the problem of VUE distance at the same time. However, this mechanism can ensure the connection reliability and quality of service (QoS), but overloading with BS or lightweight infrastructures cannot solve it. The signaling change process is a hot topic issue, too. Most literature hopes to simplify the signaling change process to reduce the latency so that handover can be perfect [16]. For handover strategy, there is much literature that focuses on the study of the QoS [17] and quality of experience (QoE) [18]. Considering the situation for MIMO: the loading of 5G is higher than LTE-A, so it cannot only use QoS for the metric to solve the problem of overloading. Hence, in this paper, we do not only consider the QoS. We also discuss the load balance between BS and lightweight infrastructures in a high-overload environment.

There are still some problems with the handover strategy in 5G. They are energy-efficiency, strange signal, data transmission rate, and latency, respectively [19]. Therefore, we consider the NR environment, and this paper will also focus on latency and resource reuse. When massive VUEs access the network, the BS/lightweight infrastructure might overload easily and impact the quality of service (QoS) or quality of experience (QoE). Therefore, the computing power needs to increase to reduce the latency. By performing this, power consumption will become a big problem with BS/lightweight infrastructure, but recently, most research has discussed the load balance with the number of VUE and power consumption [20,21,22,23]. In [20], they pointed out that growing up with a number of smartphones is a common experience; at the same time, the service requests also become more complex. Consider that the spectrum resource is limited with BS/lightweight infrastructure. It is a challenge to deploy BS/lightweight infrastructure to achieve a win–win for the operator and UE, but they only focus on throughput and signal strength. In [21], they use the roadside unit (RSU) and receive signal strength to perform the baseline for handover in VANET. These two papers show how load balance is important for BS, and they also defined the loading rate with BS. In [22], they showed that MIMO and energy-efficiency are critical in 5G. Considering the actual situation, the services required by all VUEs are different. Wu et al. proposed using the Markov chain to predict the behavior of VUE, and they also used the sleeping mechanism with lightweight infrastructure to achieve the goal of reducing the energy consumption [23]. When overloading happens, the BS will perform the scheduling again to decide the VUE priority, and it is easy to increase the delay. Therefore, the handover strategy in this paper will consider the load problem, that is, allocate VUE to the BS/lightweight infrastructure with the lower load as much as possible. It is expected to be able to reach a state where both the BS and the lightweight infrastructure can achieve load balance. At the same time, since IoV is a dynamic network environment, the fast fading caused by the user’s location and movement will affect the delay, so it will also be considered.

By predicting the VUE trajectory, we can obtain the location for the next time. This information can reduce the latency that selects the target BS/lightweight infrastructure. Most studies point out that if we collect the trajectory information and location, we can predict the VUE location to become more accurate when using deep learning or some trajectory prediction algorithm [24,25,26]. This work will define problems and goals by using load balancing, energy efficiency, user mobility, and latency. In the proposed method, first, we predict the VUE location for the next time slot and deploy the accessible candidate. Then, the hill-climbing (HC) algorithm or the simulated annealing (SA) algorithm is used to obtain the best handover strategy for the overall network performance. The contribution of this paper is as follows:

(1) Although there have been many studies on handover strategies, most literature still focuses on designing handover strategies using thresholds and *RSS*. At the same time, most literature also focuses on vertical handovers and does not consider horizontal handovers. Therefore, we consider delay time, energy efficiency, load balancing, and energy consumption and formulate it as a multi-objective optimization (MOO) problem in this paper.
(2) To solve the handover selection issue, we need to consider the real time in this paper. It is necessary to predict the location at the next time slot. Therefore, we use the angle between the vehicle and the tire to predict the location of VUE. Then, for the MOO problem, we use the HC algorithm and SA algorithm to find the best handover of the mobile management strategy. After predicting the position of the next time slot, we process handover selection based on the prediction result. Although the computing time for the heuristic algorithm cannot be ignored, it will not cause too much influence on the real-time problem.
(3) In this paper, our proposed handover of the mobile management strategy focuses on the next time slot. We predict the position of the next time slot, then process handover selection based on the prediction result. Although the heuristic algorithm still requires a long computing time, it can satisfy the real-time problem because it is a handover strategy for the next time slot.

The main layout of this paper is as follows. Section 2 is related work, and then we define the system model that some important symbol to present our problem. Section 4 is the proposed mechanism for this paper to solve the handover problem. The simulation results, analysis, and discussion will appear in Section 5. Finally, the conclusion and future work will be provided in Section 6.

## 2. Materials

There is much literature on location prediction in IoV. In [27], they used consecutive GPS location as vehicle trajectory data, which would be divided into subsets, and designed a hidden Markov model from these subsets. Although location prediction is possible through hidden Markov models and the Viterbi algorithm, they found that when the number of hidden states increases, the prediction time also increases. To solve this problem, they also proposed an enhanced Viterbi algorithm. In VANET, considering the high mobility, it is necessary to predict the location of neighboring nodes for communication. Lui et al. [28] proposed the prediction model based on Kalman filter theory, and it considers the temporal and spatial future of each node. At the same time, the use of vehicle trajectories with deep learning for location prediction has also become a topic method in recent years. In [29], they proposed a convolutional embedding model (CEM) to predict the location of the next time slot. For the embedding vector, they consider the objects, location, and time slot. The model for context was established by one-dimensional convolution. In addition, they also consider the relative order of location in the context to enhance the prediction accuracy. In [30], a location prediction model for assistance to medical vehicles was proposed, considering the best method for location prediction where the trajectory information is the only consideration. They take into account real-time traffic information and use deep belief networks and long short-term memory to achieve the goal of location prediction. There have been many studies on the trajectory-based prediction model. If the data is lacking or inaccurate, the prediction accuracy will be lower. In this paper, we consider the lack of trajectory data and the unclear real-time traffic conditions; the prediction based on the angle between the vehicle and the tire is deployed, and the computing time reduced.

According to Table 1, we can know that the QoS for handover is very important [31]. Otherwise, most literature also considers the handover trigger time or strategy with BS and small cell. In [12], they present the handover flow and trigger condition in the wireless access network. At this network architecture, the eNodeB via the S1 interface connects the mobile management entity (MME), and the interface of X2 will connect the next eNodeB. When UEs are under the covering range with the original eNodeB, the value of *RSRP* can receive the report. When UEs have moved to the edge of the service range, the *RSRP* and interference will become low. Until the *RSRP* is smaller than the threshold for handover, the handover mechanism will trigger. When the handover is triggered, the UE will disconnect from the original eNodeB when the handover is completed. From this literature, we can know the correlation between the handover trigger and *RSRP*. According to the NR, it is a HetNet architecture; in [32], they take the macrocell and femtocell into account for handover, and they also consider the channel fast fading model, *RSS*, and load balance. They proposed using the Markov chain to decide the handover strategy to solve the problem for handover. However, there is not only horizontal handover in HetNet. The vertical handover will be activated because the operator may wish to maintain the level of QoS. Goudarzi et al. [33] used the artificial bee colony—particle swarm optimization algorithm to solve the vertical handover issue to maintain the QoS; meanwhile, the UEs can access the best BS. In their work, they also consider latency, error rate, and throughput in handover strategies. Following [12,32,33], they highlight the key point metrics for designing the handover mechanism to keep the QoS.

In [34], they considered the load balance when designing the handover threshold to guarantee the *RSS*. They also reduced the probability of overload with BSs or small cells. In addition to load balance, the ping-pong effect is also a big problem with the handover. Considering the condition for triggering the handover is based on the *RSS*, if the *RSS* changes are very violent, the UEs will process the handover continuously between the two infrastructures. When the ping-pong effect occurs, in addition to the increase in latency, invalid handovers will also increase, resulting in reduced energy efficiency. To solve this problem, ref. [35] proposed using calculation of the UE waiting time in the BSs or small cells and recording the access information to find where the ping-pong effect happens at which UEs. This mechanism can reduce the redundant number of handovers to improve the network performance. In [36], they emphasized that the traditional handover method cannot be used in ultra-dense heterogeneous networks due to the number of small cells deployed. Hence, they proposed adaptive cell selection to solve the problem of handover in heterogeneous networks. First, they collect important thresholds for the current state of the environment, including RSSI, load, and speed. The speed threshold is used to decide the handover to the BSs or the small cell. If RSSI is larger than the threshold, the BSs will be selected; otherwise, choose the small cell. The RSSI is only considered when determining handover to the BSs. If it is a small cell, the load needs to be added into consideration. For candidate BSs or small cells, the cone angle is used for determination. For small cells, the best handover target in the candidate is to select the smallest cone angle. At the same time, in [37], the small cell switching mechanism in the urban environment is analyzed and discussed, and the handover of various channel models is mainly studied.

Due to the deployment of deep learning and reinforcement learning, some methods through the learning mechanism were used to select BSs or small cells and implement the handover. In [38], they investigated IoV in heterogeneous networks. This study focused on the handover mechanism, network selection, and routing. In the part of the handover, they deployed dynamic Q-learning to reduce the number of handovers. They define speed and signal strength as states and handover yes or no as action. The policy was based on an ϵ-greedy algorithm; meanwhile, in [39], they also applied deep learning-based handover methods to select the small cells. To solve the NP-hard problem of selecting small cells, they used the real situation of Los Angeles as the dataset and the feed-forward back-propagation artificial neural network as the learning model. The features for the training handover model are the angle between vehicle direction and north, longitude, latitude, and vehicle speed. In addition to using deep learning for handover prediction, in [40], they also proposed the Markov chains as a handover mechanism. Although many studies have proposed novel methods for the handover mechanism, the overall consideration is not complete. Some studies still use RSSI or distance as the basis for handovers and do not fully consider the problems caused by horizontal handovers and vertical handovers.

## 3. Problem Definition

In this section, the problem we want to solve will be discussed. At the same time, we also use the mathematical model to define the objective function. First, the network system model and the symbol defined are needed. Then, linear programming is used to present our objective.

### 3.1. System Model

**Figure 1:**
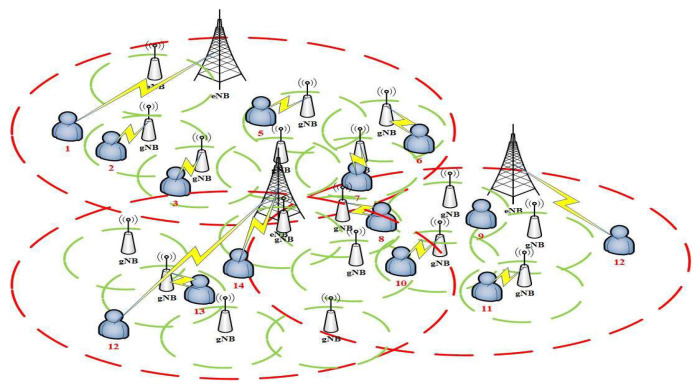
The network model.

Figure 1 is our network system model. We assume that the number of UEs is *i*, the number of BSs is *m*, and number of lightweight infrastructures is *j*. At the same time, we also define $U=\left\{u_{1},u_{2},\ldots ,u_{i}\right\}$ is the set of VUE, $B=\left\{b_{1},b_{2},\ldots ,b_{m}\right\}$ presents the set of BSs, and $C=\left\{c_{1},c_{2},\ldots ,c_{j}\right\}$ is set of the lightweight infrastructures, and we also assume i>j>m. Considering this model with the service of BSs needs to cover each lightweight infrastructure, to confirm the connection between the VUE *n* and the BS *m* or lightweight infrastructure *j*, we use $l_{n,m\vee j}$ to present it and define it as

(1) $$l_{n,m\vee j}=\left\{\begin{array}{cc} 1, & Link\\ 0, & Otherwise \end{array}\right.$$

m∨j is defined, as VUE *n* will select BS *m* or lightweight infrastructure *j*. There are three constraints for our work: (1) each VUE is mobility, and the location with start and destination are not the same; (2) if VUE can access the BS/lightweight infrastructure then they have to do it; (3) when VUE connect with BS/lightweight infrastructure, the service is activated. At the same time, all of these VUEs will send the request to the target infrastructure.

According to [41,42,43], most strategy results need evaluation through the parameters that we consider. In this paper, two category parameters should be discussed. First, we consider the speed, angle, and orientation between the vehicle and the tires for the VUE behavior. Then, we need to consider the effect when VUE accesses the network. For IoV and handover, latency is an important metric. This work considers the distance between the VUE and the handover target and the signal strength affected by fast fading, which will result in different latency. If the BS/light infrastructure is overloaded, scheduling time and queue delays need to be considered. When infrastructure is overloaded, the resource blocks provided by this infrastructure are not enough to serve all VUEs currently in this infrastructure. In this case, some VUEs may be directed to the rest of the infrastructure for service. Hence, how to balance the loading with each BS and lightweight infrastructure should be solved. Table 2 presents the important symbols and definitions for this paper.

### 3.2. Parameter Definition and Objective

According to 5G architecture, when VUE *n* finishes the register at the BS *m* or lightweight infrastructure *j*, they can connect the core network through the BS/lightweight infrastructure. Because the path and building will influence the signal to interference plus noise ratio (SINR), it and *RSRP* can weaken the signal [43]. Therefore, we used the fast-fading model to present the interference, and we defined the frequency shift by Doppler effect as Equation (2), and Equation (3) represents the fast fading influence with the channel *s*. Using Equations (2) and (3), we can know the wavelength with the Doppler effect model and fading model [44]. Equation (4) is the wavelength with Doppler effect and Equation (5) is the fast fading.

(2) $$\theta_{n,m\vee j}=\frac{v_{n}f}{c},$$

(3) $$s\left(f_{n,m\vee j}\right)=\frac{1}{\pi *\theta_{n,m}\sqrt{1-{\left(f/\theta_{n,m\vee j}\right)}^{2}}},$$

(4) $$\lambda_{n,m\vee j}=\frac{c}{s(f_{n,m\vee j})},$$

(5) $$loss_{n,m\vee j}=-10*log_{10}{\left(\frac{\lambda_{n,m\vee j}}{4\pi d_{n,m\vee j}}\right)}^{2},$$

*RSS* is still an important metric for the handover mechanism on the IoV. To know the $RSS_{n,m\vee j}$, we can use the distance between the VUE and the BS and the transmitter power for the antenna at the base station. Equation (6) shows the $RSS_{n,m\vee j}$.

(6) $$RSS_{n,m\vee j}=g_{m\vee j}^{Tx}log\left(d_{n,m}\right)*loss_{n,m\vee j}.$$

In addition to *RSS*, we define energy consumption in this section. Through the *RSS* and the RBs required for VUE services, the connection energy consumption between VUE and BSs/lightweight infrastructures can be defined as Equation (7).

(7) $$p_{n,m\vee j}^{c}=p_{m\vee j}^{overload}RB_{n,m\vee j}^{overpay}+p_{n,m\vee j}^{l},$$

(8) $$p_{n,m\vee j}^{l}=RSS_{n,m\vee j}*p_{m\vee j}^{basic-t},$$

Otherwise, the remaining energy of each BS/lightweight infrastructure is also one of the critical metrics of this paper. For the handover, to reduce the energy consumption of the connection, it may lead to the selection of the nearest BS/lightweight infrastructure for connection. Therefore, $p_{m\vee j}^{remain}$ represents the remaining energy of the BS/lightweight. Equation (10) is the total power consumption of the BS *m*/lightweight infrastructure *j*.

(9) $$p_{m\vee j}^{remain}=P_{m\vee j}^{base}-p_{n,m\vee j}^{c},$$

(10) $$p_{m\vee j}^{total-c}=\sum p_{n,m\vee j}^{c}*l_{n,m\vee j},$$

In addition to considering energy consumption, the loading for each BS/lightweight infrastructure must also be discussed in this paper because if the BS/lightweight infrastructure is selected as the handover target with a heavy load, it will lead to increased latency or decrease the QoS. $r_{n,m\vee j}^{load}$ represents the load of the BS/lightweight infrastructure when serving VUE *n*.

(11) $$r_{n,m\vee j}^{load}=\frac{RB_{n,m\vee j}^{service}*l_{n,m\vee j}}{RB_{m\vee j}^{Total}},$$

For BS/lightweight infrastructure, if there are enough resource blocks to serve the VUE, the service of VUE needs to be met as much as possible. Conversely, this paper assumes that each base station or lightweight infrastructure reserves a certain number of resource blocks. When BSs/lightweight infrastructure overloads, they can use reserve resource blocks to serve the VUE. The formula for the resource block provided by the BS/lightweight infrastructure is as (12).

(12) $$RB_{n,m\vee j}^{pro}=\left\{\begin{array}{cc} RB_{n,m\vee j}^{service}*l_{n,m\vee j} & r_{m\vee j}^{load}<r_{threshold}^{load}\\ RB_{m\vee j}^{Total}*\left(1-\sum_{n}r_{n,m\vee j}^{load}\right) & r^{load}\geq r_{threshold}^{load} \end{array}\right.$$

Consider that the latency will influence the QoS; hence, we will define the latency in this subsection. For the latency, to allow VUE to obtain enough resource blocks when the *RSS* is less than the threshold, the BSs/lightweight infrastructure will use the reserved resource blocks to avoid packet loss that causes the delay time to become longer. On the contrary, only the resource blocks required by the VUE need to be provided, so no additional latency is required. At the same time, we also consider queuing when an overload occurs. We define the $t_{n,m\vee j}$ to present the latency of VUE *n* at the BS/lightweight infrastructure, and $\alpha_{overpay}$ is the parameter of the resource block that needs to be provided additionally.

(13) $$\begin{array}{c} t_{n,m\vee j}=\left\{\begin{array}{cc} t_{n,m\vee j}^{basic}+RB_{n}^{service}*t_{n,m\vee j}^{RB}+2e^{r_{threshold}^{load}} & \\ ,RSS_{n,m\vee j}<RSS_{threshold}\\ t_{n,m\vee j}^{basic}+RB_{n}^{service}\left(1+\alpha_{overpay}\right)*t_{n,m\vee j}^{RB}+2e^{r_{threshold}^{load}} & \\ ,RSS_{n,m\vee j}\geq RSS_{threshold} \end{array}\right. \end{array}$$

In this work, $E_{n,m\vee j}$ is taken as the energy efficiency of the BS/lightweight infrastructure service VUE. Considering that energy efficiency is related to the latency, transmission energy consumption, and resource blocks provided to the VUE, the energy efficiency formula of this study is as follows Equation (14).

(14) $$E_{n,m\vee j}=\frac{RB_{n,m\vee j}^{pro}l_{n,m\vee j}}{p_{m\vee j}^{total-c}*t_{n,m\vee j}}.$$

Following the above equation, we can obtain the objective function as Equation (15), and one of the goals is to maximize energy efficiency because maximum energy efficiency means that more resource blocks must be provided by the BS/lightweight infrastructure. It will also increase the load and the delay. The MOO problem in this paper is shown in Equation (16). The $q_{n,m\vee j}$ is denoted as QoS, $\bar{E}$ represents the average energy efficiency, $\bar{t}$ is average latency, and $\bar{r}$ is denoted as average load.

(15) $$q_{n,m\vee j}=w_{1}E_{n,m\vee j}+\left(1-w_{1}\right)\left(1-{\bar{r}}_{n,m\vee j}^{load}\right).$$

(16) $$\left\{\begin{array}{cc} Maximize & \bar{E}\\ Minimize & \bar{t}\\ Minimize & \bar{r} \end{array}\right.$$

Table 3 lists the objective functions and constraints of this paper.

## 4. Method

**Figure 2:**
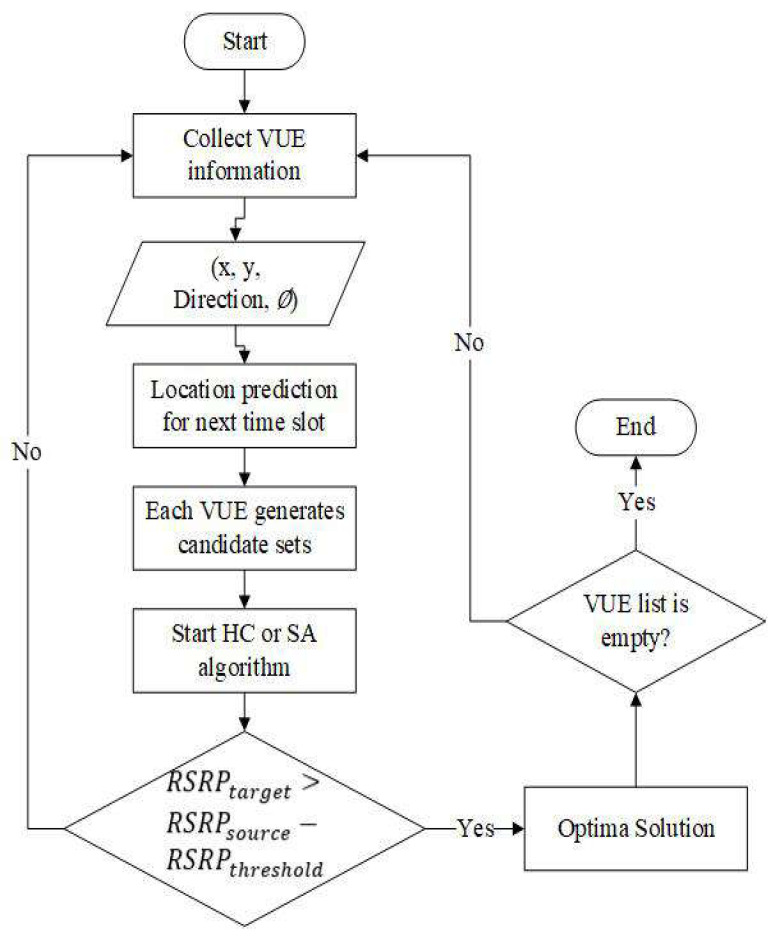
The flowchart of our proposed method.

In this paper, we want to solve the handover strategy problem for VUEs. For this situation, we propose four steps for handover. They are collecting information of VUE, predicting the location (virtual location) for the next time slot, simulating resource allocation for handover, and processing handover strategy, respectively. Following Figure 2, when VUEs access the BSs/lightweight infrastructures, it will start collecting the information and find the location of VUE. After that, the location prediction will proceed. At the same time, the MME can obtain the virtual position. Next, we need to decide which BSs/lightweight infrastructure VUE can access. The concept for this paper is that when infrastructure updates the VUE location, it also updates the candidate infrastructure. To obtain the handover strategy, we will deploy the HC and SA algorithms to find the optimal solution. According to the handover preparation period, the original infrastructure checks the *RSS* through the measurement report and decides whether to perform handover and triggers it, and we also assume that when there are no VUEs in the infrastructure, they will sleep until the other VUEs access the system.

### 4.1. Location Prediction

Considering the location of the next time for VUEs is a critical metric with handover. Therefore, how to predict the VUEs location should be discussed for the handover mechanism. To obtain the next time location for VUEs, [45] propose using the included angle with tires to predict the vehicle location, as well as the coordinates in the environment where it can be defined, according to [32,34], due to the VUEs also at the BS/lightweight infrastructure transmission range. Hence, we use the included angle between VUEs and infrastructure that predicts the next time slot location for VUEs.

We assume the angle between the vehicle and tire is known. We can define the mobility vector as $\vec{v}$. The ϕ is the included angle between $\vec{v}$ and the x-axis. Equations (17) and (18) can help us to find the next time location.

(17) $$x^{\prime }=\vec{v}+\vec{v}cos\phi ,$$

(18) $$y^{\prime }=\vec{v}+\vec{v}sin\phi .$$

### 4.2. Handover Strategy in 5G

**Figure 3:**
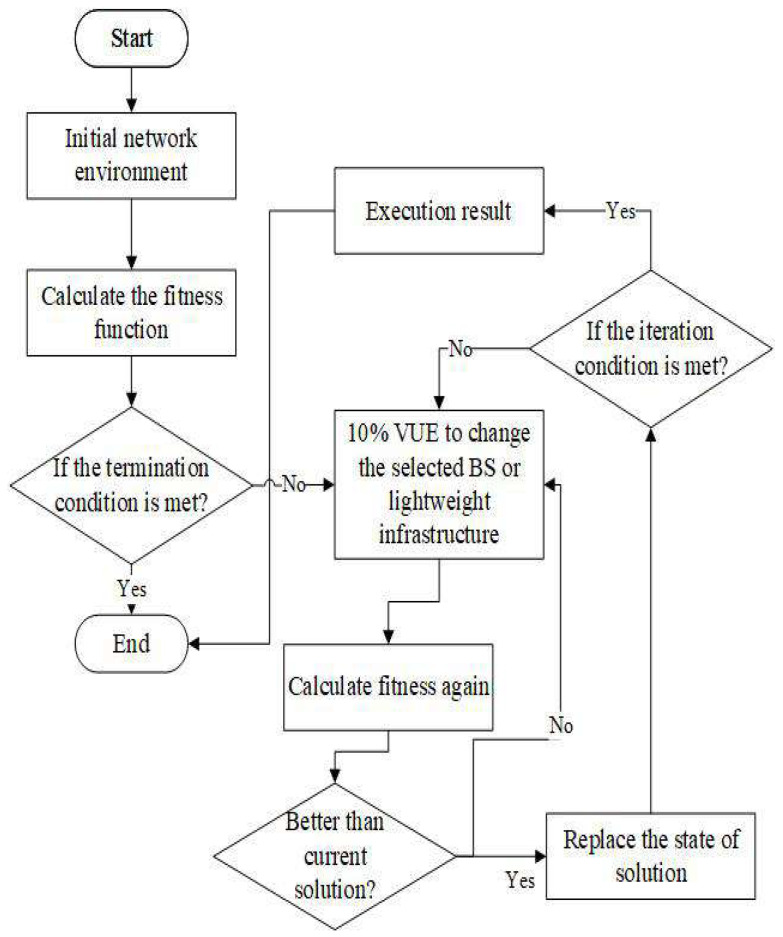
The flowchart for HC algorithm.

**Figure 4:**
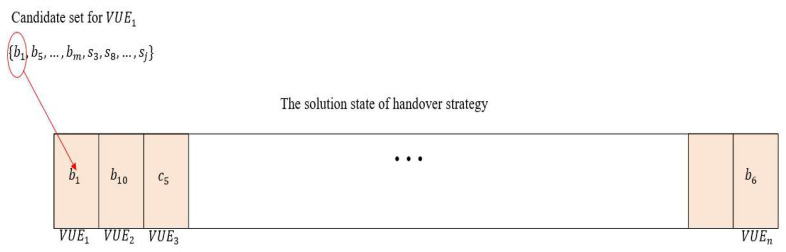
The states of the HC and SA solutions.

The HC and SA algorithm can help us find the best handover strategy for this paper. Figure 3 is the HC algorithm flowchart. To clearly explain the HC algorithm, we must define the state of the solution. Figure 4 shows the states of the HC and SA solutions in this paper. Each of these bits is denoted as VUE, and the base station or lightweight infrastructure will be selected from the candidate set as the target. We also define the initial state of the solution by choosing the nearest infrastructure. The next step is to change the solution state. We define the number of bits that we want to change as 10% at each iterative in this paper. Then, the objective function will be recalculated. If the object solution in this iteration is better than the previous solution, the new solution state will replace the old solution state and update the object solution. By processing the iterative, we may approach the optimal solution for our problem.

In addition to using the HC algorithm (Algorithm 1) to find the best handover strategy, we also use the SA algorithm to solve this problem. The SA is simulating the temperature variation to remove the impurity in metallurgy. The difference between SA and HC is the ability to escape local optima. At the HC algorithm, the new solution will replace the current one, when the new one is better than the current one. In the SA algorithm, the new solution will also replace the current solution if it is better than the current solution, but the difference is that the worse solution has the probability to replace the better solution, thereby making the solution space larger and getting rid of the local optimal solution. The probability function is

(19) $$\eta =e^{-(sol_{new}-sol)/T}.$$

**Figure 5:**
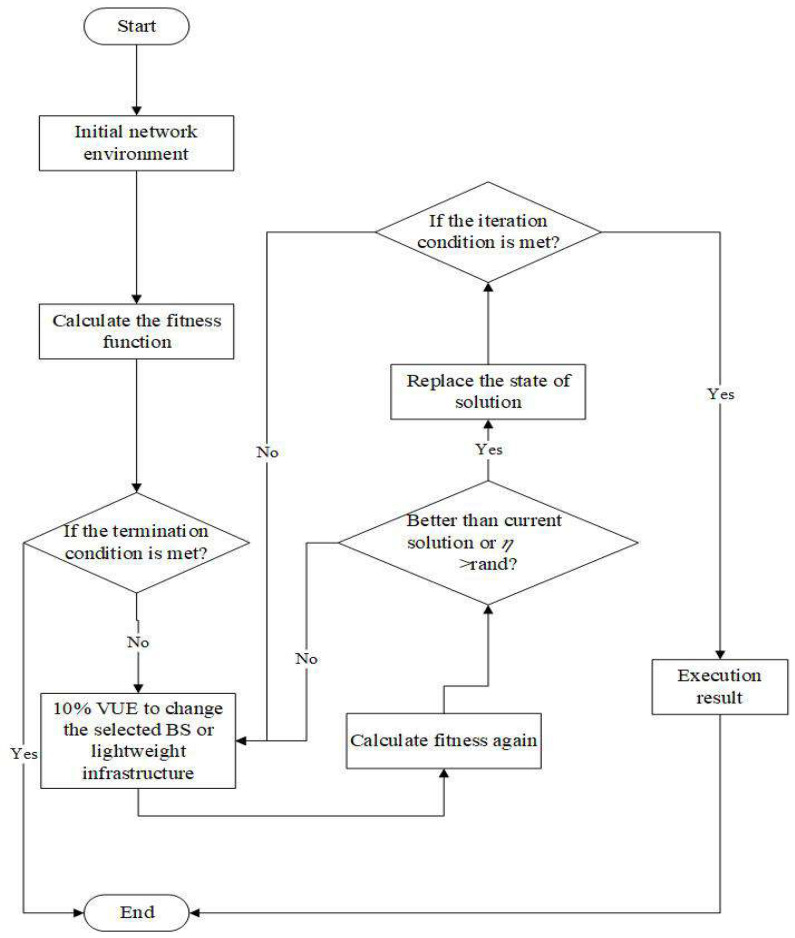
The flowchart of SA algorithm.

We define the *T* as temperature for SA. Figure 5 is the SA algorithm flowchart, and Algorithm 2 is the SA algorithm. To solve this problem, the solution state also follows the closest distance between VUE and infrastructure. The state of the solution is the same as the HC algorithm. We still choose the infrastructure nearest VUE as the initial solution state, obtain the current solution through the objective function, and define it as sol. In line 5, the SA algorithm also randomly selects 10% of the VUEs to change the infrastructure that they can access and then calculates the objective function of the new solution state, and it is defined as $sol_{new}$. Line 7 determines whether the conditions of the SA algorithm are met when $sol_{new}$ is better than sol or the probability is greater than a random value. From Equation (19), it can be known that when the temperature is lower, the probability of the $sol_{new}$ replacing the sol is smaller. Hence, we can think of it as the solution will gradually converge. If the conditions are met, the state of the current solution will replace the state of the original solution. Otherwise, the state of the solution will not change.

**Algorithm 1** Hill-Climbing-based Handover Algorithm
**Input:** Number of VUEs, *i*; Number of BSs, *m*; Number of lightweight infrastructure, *j***Output:** sol
  1:Initialize network environment  2:Generate the state of the solution following the distance between VUEs and BSs/lightweight infrastructure  3:Calculate Sol  4:
**repeat**
  5:    Randomly replace the 10% BSs or lightweight infrastructure from the state of the solution  6:    Calculate the new solution $Sol_{new}$  7:    If $Sol_{new}$>Sol  8:    Sol=$Sol_{new}$  9:    end if10:**until** iteration=max


**Algorithm 2** Simulate Anneal-based Handover Algorithm
**Input:** Number of VUEs, *i*; Number of BSs, *m*; Number of lightweight infrastructure, *j*; Temperature, *T*; η**Output:** sol
  1:Initialize network environment  2:Generate the state of the solution following the distance between VUEs and BSs/lightweight infrastructure  3:Calculate Sol  4:
**repeat**
  5:    Randomly replace the 10% BSs or lightweight infrastructure from the state of the solution  6:    Calculate the new solution $Sol_{new}$  7:    If $Sol_{new}>Sol\left|\right|\eta >random$  8:    Sol=$Sol_{new}$  9:    end if10:  T=T∗0.9711:**until** iteration=max


## 5. Results and Discussion

### 5.1. Simulation Setting

**Figure 6:**
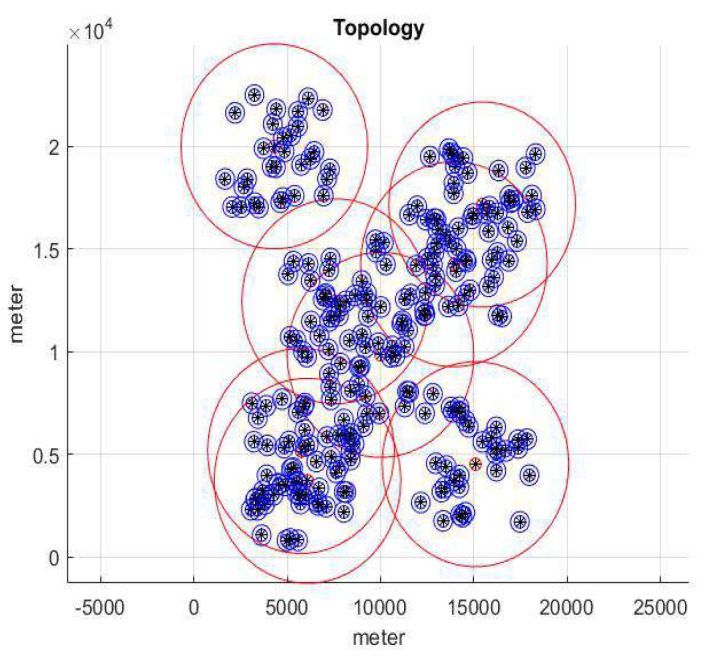
Network topology.

To verify our proposed handover issue, we use MATLAB 2017 to perform the simulation in this paper. Figure 6 represents network topology. We set the topology as 20 km × 20 km, then set the transmission power consumption for BS as 40 W, and Tx-Power is 30 dbm. Meanwhile, the transmission power consumption of lightweight infrastructure is 20 W, and Tx-Power is 46 dBm. The red area is the BS service range, and the other is the lightweight infrastructure service range. In this paper, the lightweight infrastructure is defined as the small cell (SC).

**Figure 7:**
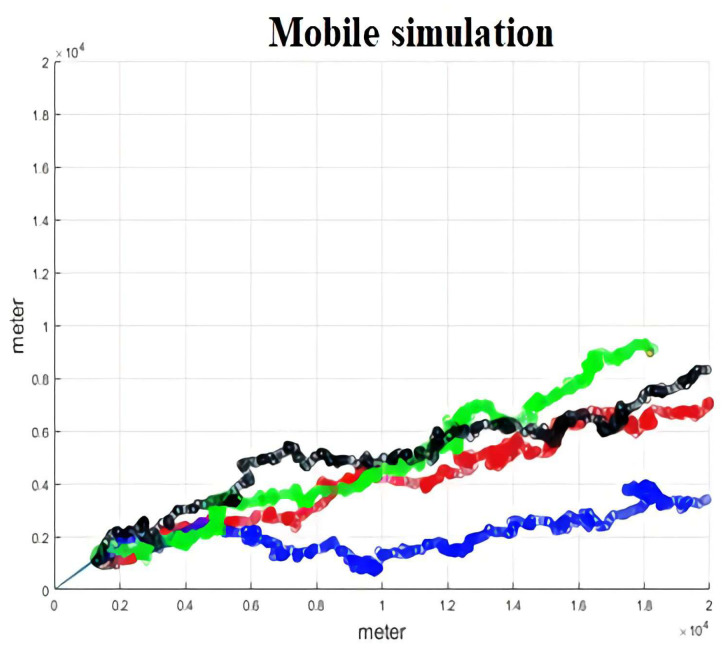
The mobile trajectory module.

In Figure 7, we use the mobile model referred to as Matlab. There are three types of mobility models: random waypoint (RWP), random direction (RD), and random walk. We add the negative angle function in the RD function to make the model more like the real world.

### 5.2. Simulation Results and Analysis

**Figure 8:**
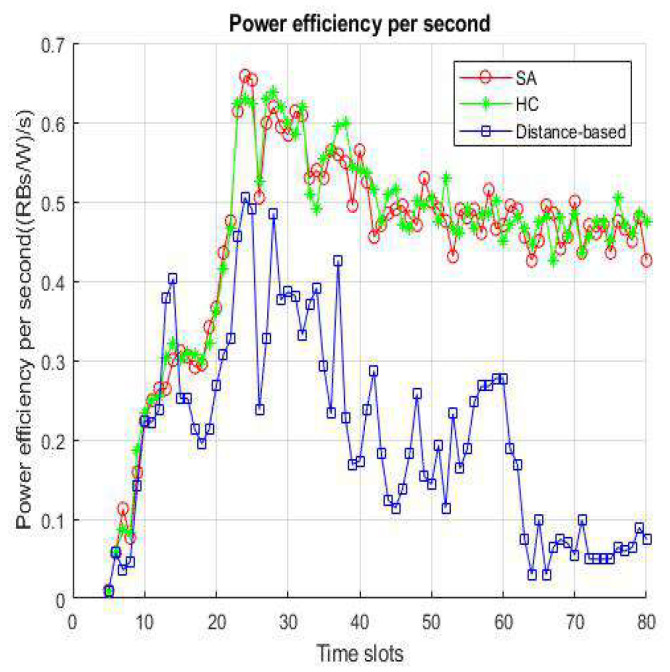
Power efficiency per time slot.

**Figure 9:**
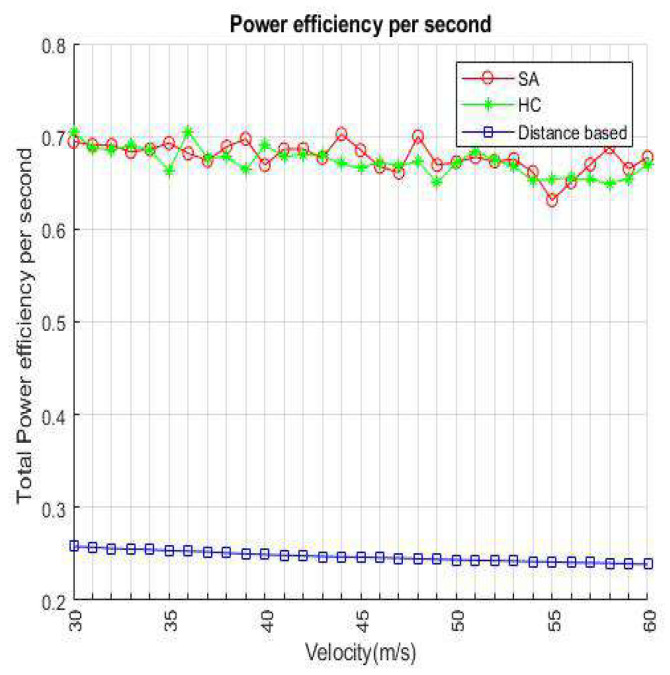
Power efficiency per velocity.

Figure 8 shows a comparison of three algorithms in continuous time for this work. Power efficiency only has a few differences when VUE keeps moving to the service range of infrastructure. Because the VUE has the unanimous original location and velocity, there are not many BS/lightweight infrastructures that VUE can choose from in the candidate set. Consider the BSs and small cells still have the possibility for overloading in this situation, SA and HC have a little higher efficiency than distance-based. When 10–13 s has passed, indicating that the VUE enters the overlapping service range between the BS and the small cell, the VUE has more access points than the previous candidates such that the BS/lightweight infrastructure will hardly be overloaded. It can be seen from the results that when the time is longer, it means that there are more choices for VUE, and the load of the BS or small cell also increases, which highlights that the method we propose is better than the distance-based handover strategy. Figure 9 shows the velocity affected for each method. We set the number of VUEs as 4500, small cells as 240, and BSs as 8 in the network environment. We only modify the parameter of velocity to a fair experiment. It represents that distance-based handover strategies are susceptible to fast fading from speed. To select the target of handover access, we use the load, energy efficiency, and latency as the basis in this work. Both HC and SA use an iterative means to choose the most suitable access BS/lightweight infrastructure. Hence, there is no evident difference between SA and HC, but we still can notice that SA loses slightly in a few cases.

**Figure 10:**
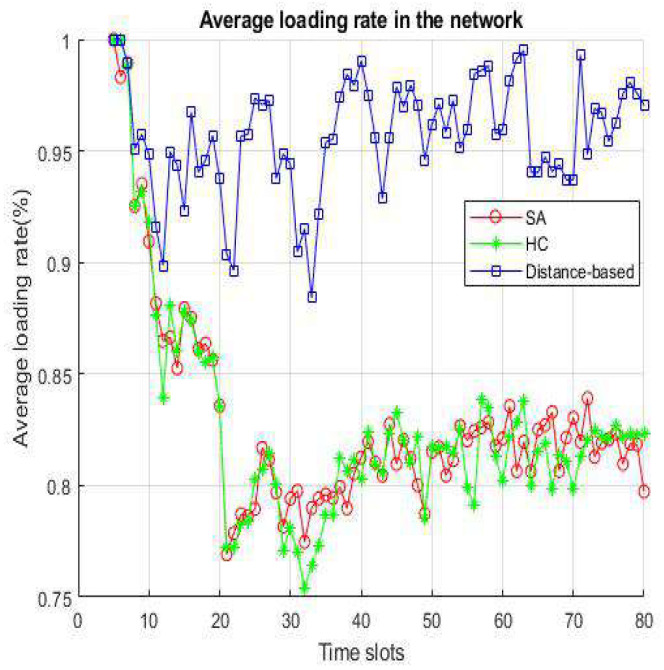
Average loading rate per time slot.

**Figure 11:**
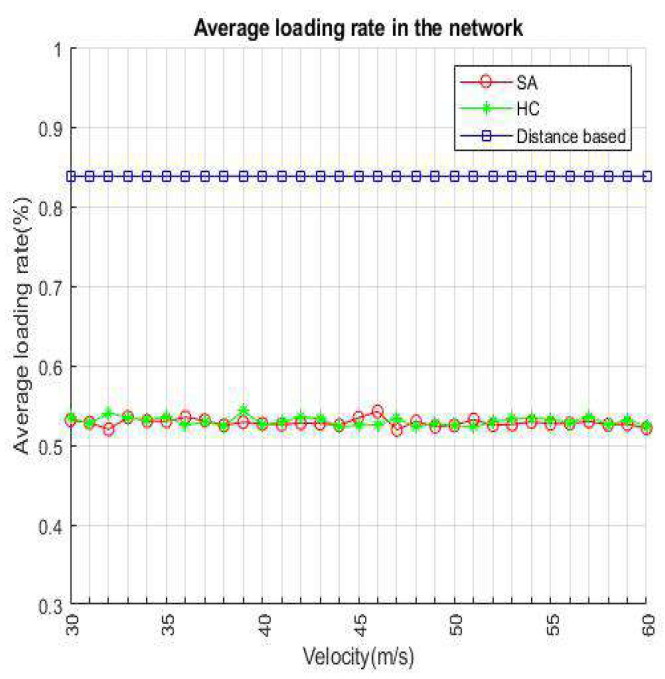
Average loading per velocity.

Figure 10 and Figure 11 represent the average loading rate per time slot and average loading rate per velocity. Both figures show that distance-based is a higher loading rate than SA and HC because in distance-based handover strategy, VUE will select an access point based on minimum distance, even if the target has a high loading rate. The difference between SA and HC is smaller than 1% in Figure 10. We can notice that SA and HC have similar results from Figure 11, because HC and SA will select the most appropriate BS/lightweight infrastructure for handover. In addition, since the distance-based handover strategy only chooses the nearest BS/lightweight infrastructure, the loading rate changes are not distinct when the speed becomes faster.

**Figure 12:**
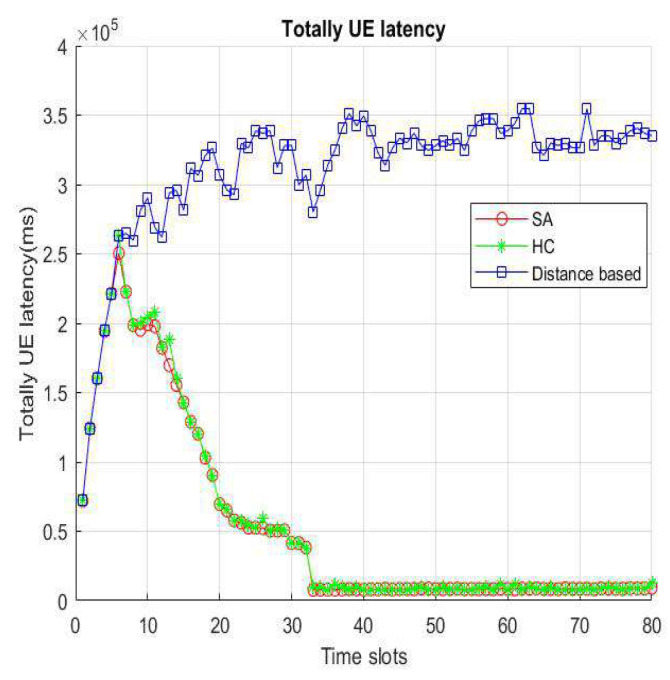
Total latency per time slot.

**Figure 13:**
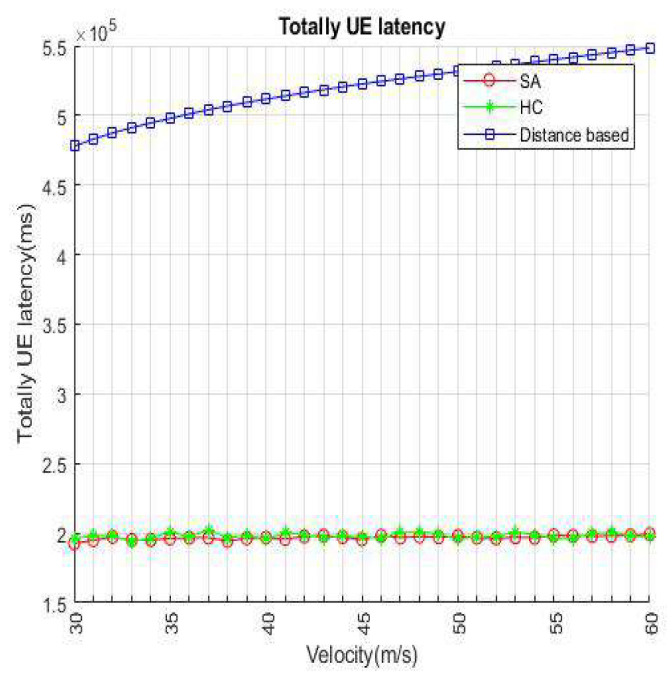
Total VUE latency per velocity.

Figure 12 and Figure 13 represent VUE latency as the sum of all VUE latency. We will consider scheduling delays and queue times when BS/lightweight infrastructure is overloaded. However, the distance-based handover strategy does not consider the impact of load; therefore, the total delay of distance-based is higher than our proposed. In the distance-based handover strategy, since all VUEs select the nearest base station/lightweight infrastructure as the access target, it is more prone to overload than the handover strategy using HC and SA, and that latency becomes large. On the other hand, since we expect to be able to load-balance the infrastructure of the entire environment, the impact of the load is considered in the handover decision. The latency will become smaller over time, and it will not change drastically with increasing speed.

**Figure 14:**
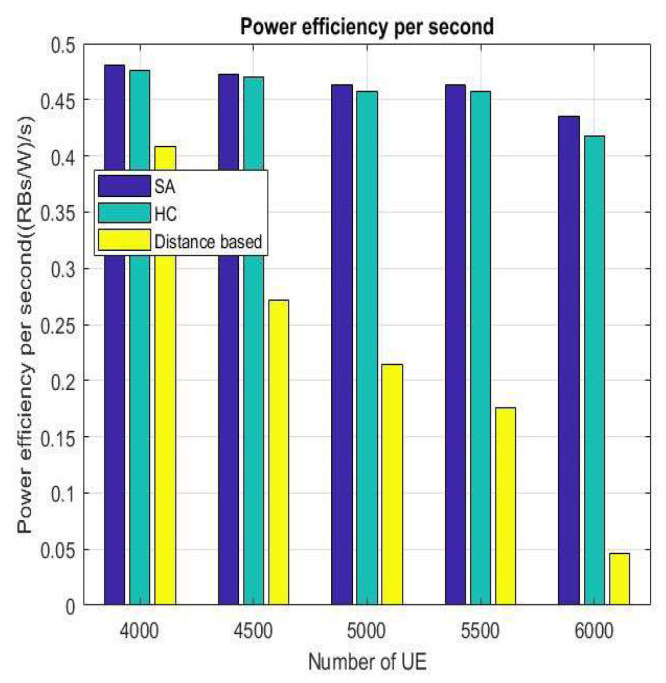
Power efficiency per second for number of different VUEs.

**Figure 15:**
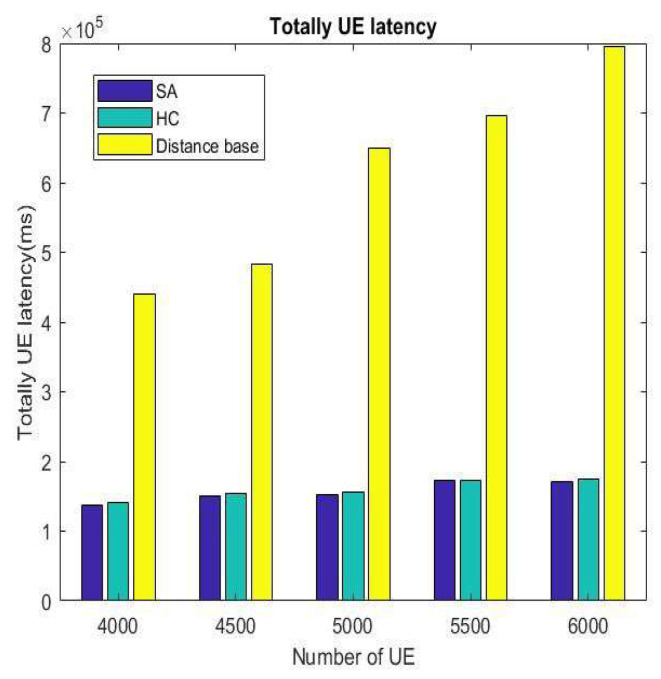
Total latency for number of different VUEs.

**Figure 16:**
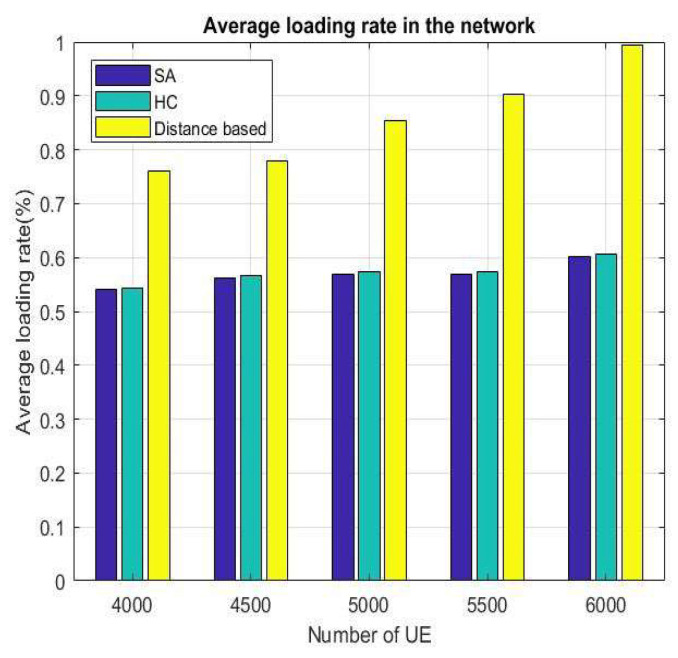
Average loading rate for number of different VUEs.

The results for power efficiency, latency, and load after changing the number of users are shown in Figure 14, Figure 15 and Figure 16, respectively. It can be seen from these three figures that when the number of VUEs increases, the distance-based handover strategy will be affected very seriously, but HC and SA do not change drastically because the mechanisms of HC and SA are very similar. They can replace the current solutions with better solutions and approach the optimal BS/lightweight infrastructure selection strategy. By doing this, we can avoid the handover strategy falling into the local optimum solution. Hence, the overall performance will be better than the distance-based handover strategy.

From the experimental results, the proposed method outperforms the distance-based handover mechanism when we adjust the number of users and the speed because we predict the location of each VUE at the next time slot first. Then, we use the HC and SA algorithms to make decisions for the select BS/lightweight infrastructure. However, it should be noted that although HC can also find the best handover strategy, it still has a greater probability of falling into the local optimum solution than SA. It is necessary to increase the number of iterations to solve this problem. For SA, because the worse solution can replace the existing solution, the solution space is bigger than HC, and it is less likely to fall into the problem of the local optimal solution.

## 6. Conclusions

In this study, in the 5G heterogeneous network environment, considering the deployment of base stations and dense lightweight infrastructure, the handover strategy of the IoV will have great challenges. Because the vehicle is mobile, frequent handovers are required. The traditional handover strategy usually only uses signal strength as a metric, which is not suitable for the 5G network environment. Therefore, this paper discusses the selection of base stations or lightweight infrastructure for vertical handover and horizontal handover of IoV. We consider energy efficiency, load, and latency for this paper. At the same time, the multi-objective optimization problem and objective function are also defined. To obtain the optimal handover strategy, we first use the angle between the vehicle and the tire to predict the location in the next time slot, then use the HC algorithm and the SA algorithm to obtain the optimal handover strategy. In experiments, we compare distance-based handover strategies, and the proposed method is better than the distance-based handover strategies because both the HC algorithm and the SA algorithm have the best ability to search for optimal solutions than the distance-based. At the same time, the SA algorithm is better than the HC algorithm because it has a mechanism to escape from the local optimal solution. We only use the angle between vehicles and tires to predict the location of VUE. Therefore, the prediction accuracy needs to improve. We will study deep learning with the trajectory method in future work. 

## Figures and Tables

**Table 1:** Mechanisms comparison.

Ref.	Year	Method	Metrics	Handover Type
[12]	2013	Based on signal strength	*RSRP* and Reference Symbol Receive Quality (RSRQ)	Horizontal
[32]	2015	Markov Chain	*RSRP* and load	Vetrical and Horizontal
[33]	2017	Hybrid the PSO and ABC	latency, throughtput, and bit error rate	Vetrical
[34]	2018	Interference-based load-dependent hysteresis handover margin	load and SINR	Vetrical and Horizontal
[35]	2018	Frequent-Handover Mitigation (FHM)	*RSRP*	Vetrical
[36]	2021	Adaptive-Cell Selection (ADA-CS)	RSSI, load, and speed	Horizontal
[38]	2021	Dynamic Q-Learning	*RSS*, and speed	Vetrical
[39]	2021	Artificial Neural Network Cell Selection (ANN-CS)	RSSI	Horizontal
[40]	2021	MarkovHandover Prediction via Markov Chain (HPMC)	*RSRP*	Horizontal

**Table 2:** The important symbols and definitions for this paper.

Symbol	Definition
$loss_{n,j\vee m}$	Fast fading model
*s*	Channel *s* with Doppler effect
θ	The frequency shift for Doppler effect
$f_{c}$	Carrier frequency
λ	The wavelength for Doppler effect
*c*	The optical speed
$E_{m\vee j}$	The Energy efficiency of BS *m*/ lightweight infrastructure *j*
$RB_{m\vee j}^{pro}$	The number of RBs for BSs/lightweight infrastructures can provide
$RB_{n}^{service}$	The number of RBs for VUE *n* requirement
$RB_{m\vee j}^{T}$	The total number of RBs for BSs/lightweight infrastructures
$g_{m\vee j}^{T_{x}}$	The antenna gain with BSs/lightweight infrastructures
$p_{m\vee j}^{ori}$	The BSs/lightweight infrastructures power already have
$p_{m\vee j}^{total-c}$	Total energy consumption
$p_{m\vee j}^{base}$	The basic energy consumption
$p_{m\vee j}^{overload}$	The power consumption for overloading
$p_{m\vee j}^{basic-t}$	The basic transmission energy for BSs/lightweight infrastructures
$t_{n,m\vee j}$	The latency for each VUE
$t_{n,m\vee j}^{basic}$	The latency for building connection
$t_{n,m\vee j}^{RB}$	The latency for RB allocate
$d_{n,m\vee j}$	The distance between VUEs and BSs/lightweight infrastructures

**Table 3:** Object function for handover.

**Maximize**	$\sum_{n}\sum_{m}\sum_{j}q_{n,m\vee j}$
**s.t.**	
	$\pi *R_{u_{i},c_{j}}^{2}\in \pi *R_{u_{i},b_{m}}^{2}$
	i>j>m,0<∀i,j,m<∞
	$\forall u_{i}\exists \left(\bar{(u_{i},c_{j})=1}\vee \bar{(u_{i},b_{m})=1}\right)$
	v=[30,210]

## Data Availability

Data is contained within the article.
